# Supplementary material for: The good, the bad and the ugly of lockdowns during Covid-19
Source: PLoS One. 2021 Jan 22;16(1):e0245546. doi: 10.1371/journal.pone.0245546 (PMC7822257; doi:10.1371/journal.pone.0245546)
Supplement: S1 Appendix — (DOCX) [file pone.0245546.s001.docx]

**S1 Appendix: Robustness checks**

**Table 4: Difference-in-Difference estimation with lagged Covid-19 related cases rather than lagged Covid-19 related deaths**

|  | **(1)** | |
| --- | --- | --- |
| **Dependent Variable: GNH** | **Coefficient** | **S.E.** |
| Lockdown*Year | -0.086* | (0.053) |
| Year | -0.177*** | (0.031) |
| Job Searches | -0.0007 | (0.0008) |
| Alcohol | -0.003*** | (0.0008) |
| Lagged Covid-19 Cases | Yes |  |
| Lagged Covid-19  Cases Squared | Yes |  |
| Lockdown F.E. | Yes |  |
| Constant | 6.59*** | (0.06) |
| N | 304 |  |
| Adjusted R2 | 0.18 |  |

Robust Standard errors in parentheses Alcohol represents “lack of beer”

* p< 0.10, ** p< 0.05, *** p< 0.01

**Table 5: Determinants of happiness during the lockdown (with "lack of tobacco" rather than alcohol)**

| **Dependent Variable: GNH** | **Coefficient** | **S.E.** |
| --- | --- | --- |
| Jobs Searches | -0.004* | (0.002) |
| Tobacco | -0.001* | (0.0006) |
| Log Tweets | -0.255** | (0.113) |
| Stay at Home Index | 0.205*** | (0.032) |
| Lagged Covid-19 Deaths | 0.013*** | (0.003) |
| Lagged Covid-19 Deaths squared | -0.002*** | (0.0007) |
| Unhappiest Week F.E. | Yes | Yes |
| Constant | 8.35*** | (0.552) |
| N | 81 |  |
| Adjusted R2 | 0.80 |  |

Robust Standard errors in parentheses

* p< 0.10, ** p< 0.05, *** p< 0.01
