# Supplementary material for: The good, the bad and the ugly of lockdowns during Covid-19
Source: PLoS One. 2021 Jan 22;16(1):e0245546. doi: 10.1371/journal.pone.0245546 (PMC7822257; doi:10.1371/journal.pone.0245546)
Supplement: Attachment — Submitted filename: Response to reviewers_PLoS_Final.docx [file pone.0245546.s003.docx]

RESPONSE TO REVIEWERS

**Manuscript ID PONE-D-20-23334**

**The good, the bad and the ugly of lockdowns during Covid-19**

We thank the reviewers for the comments. Each of these has been comprehensively addressed, as set out below (*authors' response shown in italics*). We have also made improvements throughout the paper to enhance its quality and contribution to the literature. Therefore, we believe the paper to be significantly improved as a result of the comments received from the reviewers.

**Editorial comments addressed by the authors**

**1.	Please ensure that your manuscript meets PLOS ONE's style requirements, including those for file naming. The PLOS ONE style templates can be found at**

<https://journals.plos.org/plosone/s/file?id=wjVg/PLOSOne_formatting_sample_main_body.pdf> and

<https://journals.plos.org/plosone/s/file?id=ba62/PLOSOne_formatting_sample_title_authors_affiliations.pdf>

*We thank the editor for pointing out this oversight. Subsequently, we have ensured that the PLOS ONE's style requirements are followed throughout the revised submission.*

**2.**	**Thank you for submitting the above manuscript to PLOS ONE. During our internal evaluation of the manuscript, we found significant text overlap between your submission and the following previously published works, some of which you are an author.**

<https://www.econstor.eu/bitstream/10419/217494/1/GLO-DP-0556.pdf>

**We would like to make you aware that copying extracts from previous publications, especially outside the methods section, word-for-word is unacceptable. In addition, the reproduction of text from published reports has implications for the copyright that may apply to the publications.**

**Please revise the manuscript to rephrase the duplicated text, cite your sources, and provide details as to how the current manuscript advances on previous work. Please note that further consideration is dependent on the submission of a manuscript that addresses these concerns about the overlap in text with published work.**

*We thank the editor for highlighting this issue. We refer to the email dated 26 September, which was sent by Sabine Henderson as a response to our explanation offered regarding the overlap in text. Ms Henderson agreed that since our manuscript is i) posted on EconStor only as a discussion paper, ii) has not been previously peer-reviewed or iii) formally published in a journal, that there is no need to edit our submission to PLOS ONE to resolve any of the text overlaps. If the editor wishes to see the email, we will be more than happy to supply a copy from our side.*

**3.**	**We note that you have indicated that data from this study are available upon request. PLOS only allows data to be available upon request if there are legal or ethical restrictions on sharing data publicly. For information on unacceptable data access restrictions, please see** <http://journals.plos.org/plosone/s/data-availability#loc-unacceptable-data-access-restrictions>.

*We thank the editor for pointing out the issue regarding the sharing of our data. Subsequently, we uploaded the data to the appropriate repository.*

**4.	In your revised cover letter, please address the following prompts:**

a) **If there are ethical or legal restrictions on sharing a de-identified data set, please explain them in detail (e.g., data contain potentially identifying or sensitive patient information) and who has imposed them (e.g., an ethics committee). Please also provide contact information for a data access committee, ethics committee, or other institutional body to which data requests may be sent.**

*We have indicated that there are no ethical or legal restrictions on sharing the dataset.*

b) **If there are no restrictions, please upload the minimal anonymized data set necessary to replicate your study findings as either Supporting Information files or to a stable, public repository and provide us with the relevant URLs, DOIs, or accession numbers. Please see** [**http://www.bmj.com/content/340/bmj.c181.long**](http://www.bmj.com/content/340/bmj.c181.long) **for guidelines on how to de-identify and prepare clinical data for publication. For a list of acceptable repositories, please see** <http://journals.plos.org/plosone/s/data-availability#loc-recommended-repositories>.

**We will update your Data Availability statement on your behalf to reflect the information you provide.**

*As there are no restrictions on sharing the data, we have done the necessary and uploaded our dataset to the appropriate repository.*

**5.	Thank you for stating the following financial disclosure:**

**"The funders had no role in study design, data collection and analysis, decision to publish, or preparation of the manuscript."**

**At this time, please address the following queries:**

a) **Please clarify the sources of funding (financial or material support) for your study. List the grants or organizations that supported your study, including funding received from your institution.**

*We like to point out that the grants received from our institutions which supported our study has been listed. They are the University of Johannesburg, South Africa and the Auckland University of Technology, New Zealand.*

b) **State what role the funders took in the study. If the funders had no role in your study, please state: "The funders had no role in study design, data collection and analysis, decision to publish, or preparation of the manuscript."**

*We made the necessary corrections and stated that "The funders had no role in study design, data collection and analysis, decision to publish, or preparation of the manuscript."*

c) **If any authors received a salary from any of your funders, please state which authors and which funders.**

*The following authors received salaries from their institutions, who were also the funders of the research.*

1) *Prof Talita Greyling: University of Johannesburg via the University Research Fund.*
2) *Dr Stephanie Rossouw: Auckland University of Technology via the Faculty for Business, Economics and Law.*
d) **If you did not receive any funding for this study, please state: "The authors received no specific funding for this work."**

**Please include your amended statements within your cover letter; we will change the online submission form on your behalf**

*Thank you for this comment, we updated the cover letter with the amended statements.*

**6.	PLOS requires an ORCID iD for the corresponding author in Editorial Manager on papers submitted after 6 December 2016. Please ensure that you have an ORCID iD and that it is validated in Editorial Manager. To do this, go to 'Update my Information' (in the upper left-hand corner of the main menu), and click on the Fetch/Validate link next to the ORCID field. This will take you to the ORCID site and allow you to create a new iD or authenticate a pre-existing iD in Editorial Manager.**

*Thank you for this comment, we updated the ORCID ID of the corresponding author.*

**Reviewer comments addressed by the authors**

**Reviewer #1:**

**Thank you for giving me the opportunity to review your paper.**

**1.1	The paper attempted to validate the data source by comparing the gross national happiness index to other country circumstances. However, the happiness for a population can't measured by such a method! it should be based on scientific background.**

*We thank the reviewer for this comment. However, we would like to point out that our main aim was to analyze the causal effect of a lockdown and the determinants of happiness during a lockdown, in an extreme country case. Thus, the aim was not to validate the happiness measure, as such. We believe it has been established in previous literature that measures derived from Big Data, can accurately measure happiness/sentiment, emotions and well-being, by making use of Natural Language Processing and sentiment analysis. Please see the below references:*

1. *Algan, Y., Beasley, E., Guyot, F., Higad, K., Murtin, F., & Senik, C. (2015). Big Data Measures of Well-Being: Evidence from a Google Well-Being Index in the U.S. SciencesPo Working Paper 2015.*
2. *Bellet, C. & Frijters, P. (2019). Big data and well-being. In World Happiness Report 2019, chapter 6. Edited by John F. Helliwell, Richard Layard, and Jeffrey D. Sachs. Associate Editors, Jan-Emmanuel De Neve, Haifang Huang, Shun Wang, and Lara B. Aknin.*
3. *Brodeur, A., Clark, A. E., Fleche, S. & Powdthavee, N. (2020). Assessing the impact of the coronavirus lockdown on unhappiness, loneliness, and boredom using Google Trends. arXiv preprint arXiv:2004.12129. 2020 25 April.*
4. *Dodds, P. S. & Danforth, C. M. (2010). Measuring the Happiness of Large-Scale Written Expression: Songs, Blogs, and Presidents. Journal of Happiness Studies, 11: 441–456.*
5. *Dodds, P. S., Harris, K. D., Kloumann, I. M., Bliss, C. A. & Danforth, C. M. (2011). Temporal patterns of happiness and information in a global social network: Hedonometrics and twitter. PLoS ONE. DOI:10.1371/journal.pone.0026752.*
6. *Ford, M.T., Jebb, A. T., Tay, L. & Diener, E. (2018). Internet Searches for Affect-Related Terms: An Indicator of Subjective Well-Being and Predictor of Health Outcomes across U.S. States and Metro Areas. Applied Psychology: Health and Well-being, 10(1): 3–29.*
7. *Hamermesh, D. S. (2020). Lockdowns, loneliness and life satisfaction. National Bureau of Economic Research; 2020 16 April.*
8. *Rossouw, S., Greyling, T., Adhikari, T. & Morrison, P. S. (2020). Markov switching models for happiness during a pandemic: The New Zealand experience. GLO Discussion Paper, No. 573, Global Labor Organization (GLO), Essen.*
9. *Rossouw, S. & Greyling, T. (2020). Big Data and Happiness. Invited chapter for the Handbook of Labor, Human Resources and Population Economics. Edited by Klaus F. Zimmermann.*

**1.2	The manuscript is mixed in most the sections. As the result should stands alone then the discussion comes to explain the results and link it with the earlier literature. Also, discussing the strengths or defending the limitation should lie under the discussion section not before.**

*We thank the reviewer for this comment. However, we believe that various disciplines approach the writing of research papers differently. As we are editors ourselves of interdisciplinary journals, we notice that there are significant variations in the writing style of the manuscripts we process. For example, we have noticed that often in Psychology, the method, as mentioned above by yourself, is used. However, this is not the norm in Economics or Well-being studies. Therefore, we follow the generally accepted method when writing research papers in Economics and Well-being studies.*

**Reviewer #2:**

**This is a very interesting article covering an often overlooked factor in society - happiness. Using Twitter activity as a proxy, it seeks to assess the impact of the prevailing covid-19 pandemic lockdowns on happiness of the general population in a nation with pre-existing societal well-being challenges. It is thus both timely and pioneering in its approach as well as meet for the moment and useful for guiding public policy.**

**Further, the authors employ a unique dataset and did well in providing explanations for their analytic decisions. These would help inform the interpretations by the readers, post-publication, and add to the analysis pool in general.**

*We thank the reviewer for this kind and generous review of our study.*

**1) Major Comments**

a) **The paper would be enriched if the graph visualizing the 'inverted U-shape relationship' were added as this would provide some clarity about the variables on the axes.**

*We thank the reviewer for this suggestion and agree that adding the graph would add value to the paper. Subsequently, we added the graph showing the inverted U-shaped relationship between happiness and Covid-19 deaths (please see figure 4 on page 19).*

b) **Moreover, the authors should comment on whether there might be other factors at play in/confounders to this relationship between happiness and COVID-19 deaths? The duration of the lockdown presumably aligns with the rising number of deaths and could perhaps impact happiness.**

*We thank the reviewer for raising this point. After giving it significant thought, we concluded that there might be confounding factors that can affect the inverted U-shaped relationship between happiness and lagged Covid-19 deaths. For example, social relationships. It could be that initially, families were happy because they had more time to spend with their loved ones. Still, as the lockdown period prolonged, it is plausible that these initial higher happiness levels decreased due to intensified family social relationships. However, in further studies, it was found that the longer the lockdown period lasted, the more satisfied/happy people were staying at home. Listing various reasons, amongst others was stronger social relationships with their families. Furthermore, to test the inverted U-shaped relationship, we also included and excluded other covariates and found that the reported relationship between lagged Covid-19 deaths and happiness remained. Therefore, we believe this result is robust.*

c) **Figures should be placed properly in the body of the manuscript and not at the end.**

*We thank the reviewer for this suggestion. Unfortunately, this is out of our control. We followed PLOS ONE's manuscript style requirements throughout the formatting of the manuscript, which requires that the figures should be submitted separately. The editorial management system then automatically places these figures at the end of the manuscript.*

d) **Please include the units (e.g. New Daily Deaths per million) for each variable in Table 1.**

*We thank the reviewer for the comment. Subsequently, all correction was made.*

e) **When reporting results, could the authors please indicate the relevant p-values in the text and not just in the tables?**

*We thank the reviewer for this suggestion. Subsequently, all the p-values were added in-text as well.*

f) **Could the authors please indicate the particular (qualitative) methodology adopted for the analysis of Tweets mentioned where the paper states "On analyzing the contents of the tweets, we find the following. South Africans are wary...". Please state how many tweets were assessed and also clarify if it was the research team itself that did this analysis.**

*We extract* ***all tweets*** *made by South Africans per day. We want to invite the reviewer to look at our website,* <https://gnh.today>*. For South Africa, we receive between 60 000 to 80 000 tweets per day. Using Big Data methods, we can determine the topics which were tweeted about most. Based on these, we can sort data and then we systematically read through tweets (thousands of tweets) to understand what drives the sentiment. We, as a team did all these analyses ourselves.*

g) **In saying "Also, people in the suburbs seem to be more colloquial [Should this read 'convivial'?], with strangers greeting one another.", could the authors mention if the strangers were meeting in person outside their homes during the lockdown or online?**

*We thank the reviewer for pointing out this oversight as we did mean 'convivial' and not 'colloquial'. Subsequently, we corrected our error and added that it was outside their homes as people took short walks through their neighbourhoods.*

h) **Could the authors please include some limitations to the study at an appropriate position/in the conclusion section.**

*We thank the reviewer for pointing out this oversight. Subsequently, all limitations have been acknowledged at the end of the conclusions section.*

**2) Minor Comments**

a) **In the introduction, the authors mention "what matters most"; a brief clarification of what it is that matters most would be helpful.**

*We thank the reviewer for this comment. By saying 'what matters most,' we mean nothing more than those factors that significantly influence happiness. Subsequently, we added this in brackets after the first mention in the introduction section.*

b) **The authors also repeatedly make mention of 'Big Data'; a sentence or two explaining what constitutes Big Data would also be useful.**

*We thank the reviewer for pointing out this oversight. We added a couple of sentences under section 2.3 before discussing the studies that utilized various Big Data methods.*

c) **In section 2.1, could the authors please clarify what well-being in the South African context comprises? What are the indicators/dimensions they consider besides income?**

*We thank the reviewer for this comment. However, we wish to point out that the reason for adding the paragraph under section 2.1 is to illustrate that South Africa is an extreme country case and not to highlight the dimensions/indicators under investigation. Additionally, in section 3.2.2, we state the dimensions/indicators that we consider. We selected the relevant indicators from the tweets. We determined that the main indicators fall under economic uncertainty, the prohibition of the sale of alcohol and tobacco, the stay-at-home orders and the Covid-19 pandemic itself (noting that these are significantly different from those indicators during normal times).*

*In saying the above, we did expand (and change) the paragraph under section 2.1 to highlight the reason for its inclusion better.*

d) **In the first sentence of Section 2.3 'policy' should perhaps read 'policy-makers'.**

*We thank the reviewer for pointing out this oversight. Subsequently, the error was corrected.*

e) **Although the authors state that 'Yes - all data are fully available without restriction' and 'Data can be provided separately' I did not find any underlying data within the manuscript or as an addendum. Perhaps this might be due to the statement 'Tick here if your circumstances are not covered by the questions above and you need the journal's help to make your data available.; Tick here if the URLs/accession numbers/DOIs will be available only after acceptance of the manuscript for publication so that we can ensure their inclusion before publication.' The reason notwithstanding, the absence of the underlying data makes me unable to comment on it in depth. I thus limit my comments to the data contained in the manuscript.**

*We sincerely apologize for not uploading the data upon the first submission. All data has subsequently been uploaded to the correct PLOS ONE repository.*

**Reviewer #3**:

**A very novel topic, with a very sound methodological analysis. Interesting findings; but I am concerned about the suggestions to reinstate the sale of alcohol and tobacco in order to increase happiness. Not only due to their impacts on public health, but also because the reported finding about them could be due to a confounding factor with social gatherings (where these items are usually consumed).**

a) **I would suggest delving deeper about this in the discussion and I encourage you to align a message about responsible consumption of these substances.**

*We thank the reviewer for pointing this out. Subsequently, we have added that we advocate for responsible alcohol consumption and acknowledged that the restrictions on social gatherings could confound the impact of lack of alcohol. Please see paragraph 5 in the conclusions section.*

b) **While revising your submission, please upload your figure files to the Preflight Analysis and Conversion Engine (PACE) digital diagnostic tool,** [**https://pacev2.apexcovantage.com/**](https://pacev2.apexcovantage.com/)**. PACE helps ensure that figures meet PLOS requirements. To use PACE, you must first register as a user. Registration is free. Then, login and navigate to the UPLOAD tab, where you will find detailed instructions on how to use the tool. If you encounter any issues or have any questions when using PACE, please email PLOS at figures@plos.org. Please note that Supporting Information files do not need this step.**

*We thank the reviewer for this suggestion. Subsequently, on resubmission, the above method was followed.*
